# Global and Chinese trends in acute myeloid leukemia burden (1990–2021): a comprehensive analysis based on the GBD study

**DOI:** 10.3389/fmed.2025.1629111

**Published:** 2025-09-05

**Authors:** Xiaojing Lu, Xin Peng, Yunxiang Zhang, Hongming Zhu, Yu Zheng, Yue Sun, Heyu Ji, Junmin Li, Haijiao Jin, Xiaoyang Li

**Affiliations:** ^1^Shanghai Institute of Hematology, State Key Laboratory of Medical Genomics, National Research Center for Translational Medicine at Shanghai, Ruijin Hospital Affiliated to Shanghai Jiao Tong University School of Medicine, Shanghai, China; ^2^Department of Medical Affairs, Ruijin Hospital Affiliated to Shanghai Jiao Tong University School of Medicine, Shanghai, China; ^3^Department of Nephrology, Ren Ji Hospital Affiliated to Shanghai Jiao Tong University School of Medicine, Shanghai, China

**Keywords:** acute myeloid leukemia, Global Burden of Disease, epidemiology, mortality, DALYs, risk factors, China

## Abstract

**Background:**

Acute myeloid leukemia (AML) poses a significant global health burden. This study evaluates long-term trends in AML burden from 1990 to 2021, focusing on global and Chinese patterns using Global Burden of Disease (GBD) data.

**Methods:**

We extracted AML-related mortality, incidence, prevalence, and disability-adjusted life years (DALYs) from the GBD 1990–2021 dataset. Age-standardized rates (ASRs) were analyzed using Joinpoint regression to calculate annual percentage change (APC) and average APC (AAPC). Contributions of smoking, high body mass index (BMI), and occupational exposures were also evaluated.

**Results:**

Globally, AML deaths rose by 73.8%, with a 17.45% increase in ASMR. Conversely, China's ASMR declined by 14.76%. DALYs showed a global AAPC of −0.83%, with a sharper decline in China (−2.40%). Males and older adults (>65 years) bore a disproportionate burden. Smoking remained the top risk factor, while high BMI showed the fastest growth in attributable burden.

**Conclusion:**

While AML's absolute burden is increasing worldwide, age-adjusted metrics are stabilizing or declining in China, likely due to healthcare improvements. Targeted prevention, risk control, and geriatric-oriented AML strategies are urgently needed.

## 1 Introduction

Acute myeloid leukemia (AML) is a heterogeneous group of hematological malignancies characterized by clonal expansion of abnormal myeloid progenitor cells in the bone marrow and peripheral blood, leading to hematopoietic dysfunction and bone marrow failure. As the most common acute leukemia in adults, AML remains a significant global health challenge with high mortality rates despite therapeutic advances (1, 2).

The epidemiological characteristics of AML show marked variations across regions, age groups, and genders. Understanding these patterns is crucial for health planning, resource allocation, and developing targeted interventions. While previous studies have documented AML epidemiology in specific regions or time periods, comprehensive analyses of global trends over longer timeframes remain limited (3, 4).

As the world's most populous country, China has experienced rapid population aging and significant changes in its healthcare system over the past few decades, making it an important context for analyzing AML burden trends. Comparing Chinese trends with global data helps reveal the impacts of demographic transitions, socioeconomic development, and healthcare system changes on AML outcomes (5).

This study aims to conduct a comprehensive analysis of global and Chinese AML burden from 1990 to 2021 using GBD study data. We focus on analyzing temporal trends in mortality, incidence, prevalence, and DALYs, stratified by age and gender, to identify patterns and potential determinants of burden changes. Through Joinpoint regression analysis, we identify significant trend change points and quantify the rate of change in different periods, providing deeper insights into the evolution of AML burden over the past three decades (6). To our knowledge, this is the first study to simultaneously integrate Joinpoint regression and risk factor–attributable EAPC to compare long-term AML burden between China and global data.

## 2 Methods

### 2.1 Data source

This study utilizes data provided by the Global Burden of Disease (GBD) study (https://vizhub.healthdata.org/gbd-results/). The GBD study provides comprehensive global estimates of mortality, incidence, prevalence, disability-adjusted life years (DALYs), years of life lost (YLLs), and years lived with disability (YLDs) for numerous diseases and injuries, including AML. The methodology of the GBD study has been described in detail in the literature (6). We analyzed global and China-specific AML burden estimates from 1990 to 2021.

### 2.2 Burden indicators

We extracted the following AML burden indicators:

- Mortality: death counts and age-standardized mortality rates
- Incidence: new case counts and age-standardized incidence rates
- Prevalence: prevalent case counts and age-standardized prevalence rates
- DALYs: DALY counts and age-standardized DALY rates

Age-standardized rates (ASRs) were calculated based on the GBD standard population and expressed per 100,000 population. This method accounts for differences in age structure between populations and over time, enabling more effective comparisons.

### 2.3 Statistical analysis

#### 2.3.1 Trend analysis

We analyzed trends in both absolute numbers and age-standardized rates of AML burden indicators from 1990 to 2021 and calculated the percentage change between the beginning and end of the study period.

#### 2.3.2 Joinpoint regression analysis

To identify significant changes in AML burden trends, we conducted Joinpoint regression analysis on the ASR data. This method identifies statistically significant changes in time series data and estimates trends over different time segments.

We used the following log-linear model for analysis: ln(y) $=\beta_{0}+\beta_{1}\text{x}+\delta_{1}{\left(\text{x}-\tau_{1}\right)}^{+}+\delta_{2}{\left(\text{x}-\tau_{2}\right)}^{+}+$... $+\delta_{\text{n}}{\left(\text{x}-\tau_{\text{n}}\right)}^{+}+\varepsilon$

- ln(y) is the natural logarithm of the age-standardized rate
- x is the calendar year
- τ1, τ_2_,..., τ_n_ are the joinpoint years
- (x–τ_i_)^+^ equals (x–τ_i_) when (x–τ_i_) > 0 and 0 otherwise
- β0 is the intercept, β1 is the slope of the first time segment, and δ_i_ represents the difference in slopes between adjacent time segments
- ε is the random error term

Joinpoint models were selected using Bayesian Information Criterion (BIC) minimization, with significance assessed via permutation tests (α = 0.05). Model parameters included a maximum of three joinpoints and a minimum interval of 2 years between joinpoints, as determined by Bayesian Information Criterion (BIC) minimization following software defaults. Sensitivity testing with alternative joinpoint limits yielded consistent trend patterns.

#### 2.3.3 Risk factor attribution analysis

We analyzed DALYs and deaths attributable to key modifiable risk factors for AML, including smoking, high BMI, and occupational carcinogens. Attributable burden was summarized by age group, sex, and year. Estimated Annual Percentage Change (EAPC) was computed using log-linear regression to characterize long-term trends in attributable burden.

## 3 Results

### 3.1 Overall AML burden trends

#### 3.1.1 Mortality

**Figure 1:**
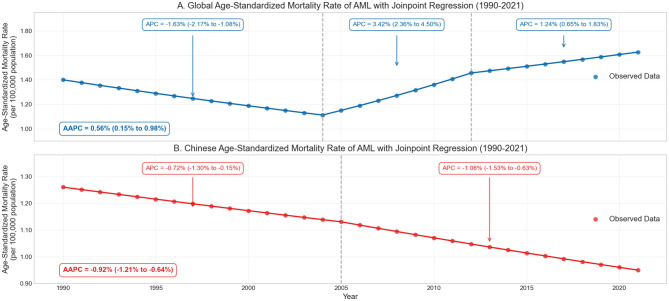
Trends in acute myeloid leukemia (AML) mortality and age-standardized mortality rates (ASMR) globally and in China from 1990 to 2021. Joinpoint regression analysis highlights trend inflection points, showing increasing global ASMR **(A)** and a contrasting declining trend in China **(B)**.

The absolute number of global AML deaths steadily increased from 74,918 in 1990 to 130,189 in 2021, representing a 73.8% increase over 32 years. However, Joinpoint regression analysis revealed that the age-standardized mortality rate (ASMR) changed through three distinct phases: 1990–2004 (APC = −1.63%, 95% CI: −2.17 to −1.08%, *p* < 0.001), 2004–2012 (APC = 3.42%, 95% CI: 2.36 to 4.50%, *p* < 0.001), and 2012–2021 (APC = 1.24%, 95% CI: 0.65 to 1.83%, *p* < 0.001). The overall AAPC for the entire study period was 0.56% (95% CI: 0.15 to 0.98%, *p* = 0.008), with ASMR increasing from 1.40/100,000 in 1990 to 1.65/100,000 in 2021, a 17.45% increase (Figure 1).

In contrast, AML deaths in China remained relatively stable, increasing slightly from 14,853 in 1990 to 15,311 in 2021 (3.1% increase), accounting for only 11.8% of global deaths, significantly lower than China's share of the global population (~18%). Joinpoint analysis showed that China's ASMR exhibited two declining phases: 1990–2005 (APC = −0.72%, 95% CI: −1.30 to −0.15%, *p* = 0.016) and 2005–2021 (APC = −1.08%, 95% CI: −1.53 to −0.63%, *p* < 0.001), with an overall AAPC of −0.92% (95% CI: −1.21 to −0.64%, *p* < 0.001). The ASMR decreased from 1.26/100,000 in 1990 to 1.08/100,000 in 2021, a decline of 14.76% (Table 1).

**Table 1:** Summary of AML burden metrics globally and in China, 1990 vs. 2021.

**Metric**	**Global 1990**		**Global 2021**		**China 1990**		**China 2021**	
	**Number**	Rate^*^	**Number**	Rate^*^	**Number**	Rate^*^	**Number**	Rate^*^
Mortality	74,918	1.40	130,189	1.65	14,853	1.26	15,311	1.08
Incidence	79,373	1.49	144,646	1.83	15,309	1.30	17,835	1.25
Prevalence	122,732	2.30	204,430	2.59	25,514	2.17	24,172	1.70
DALYs	3,342,913	62.68	4,135,056	52.40	851,931	72.41	548,555	38.56
Percentage change in mortality rate (1990–2021)	+17.45%				−14.76%			
Percentage change in DALY rate (1990–2021)	−16.40%				−46.75%			

^*^Rates are age-standardized per 100,000 population.

The opposing trends between China and global ASMR reflect significant improvements in China's healthcare accessibility and quality, particularly in leukemia diagnostic and treatment capabilities, as well as possible reductions in exposure to environmental risk factors such as benzene and ionizing radiation (7, 8).

#### 3.1.2 Incidence and prevalence

**Figure 2:**
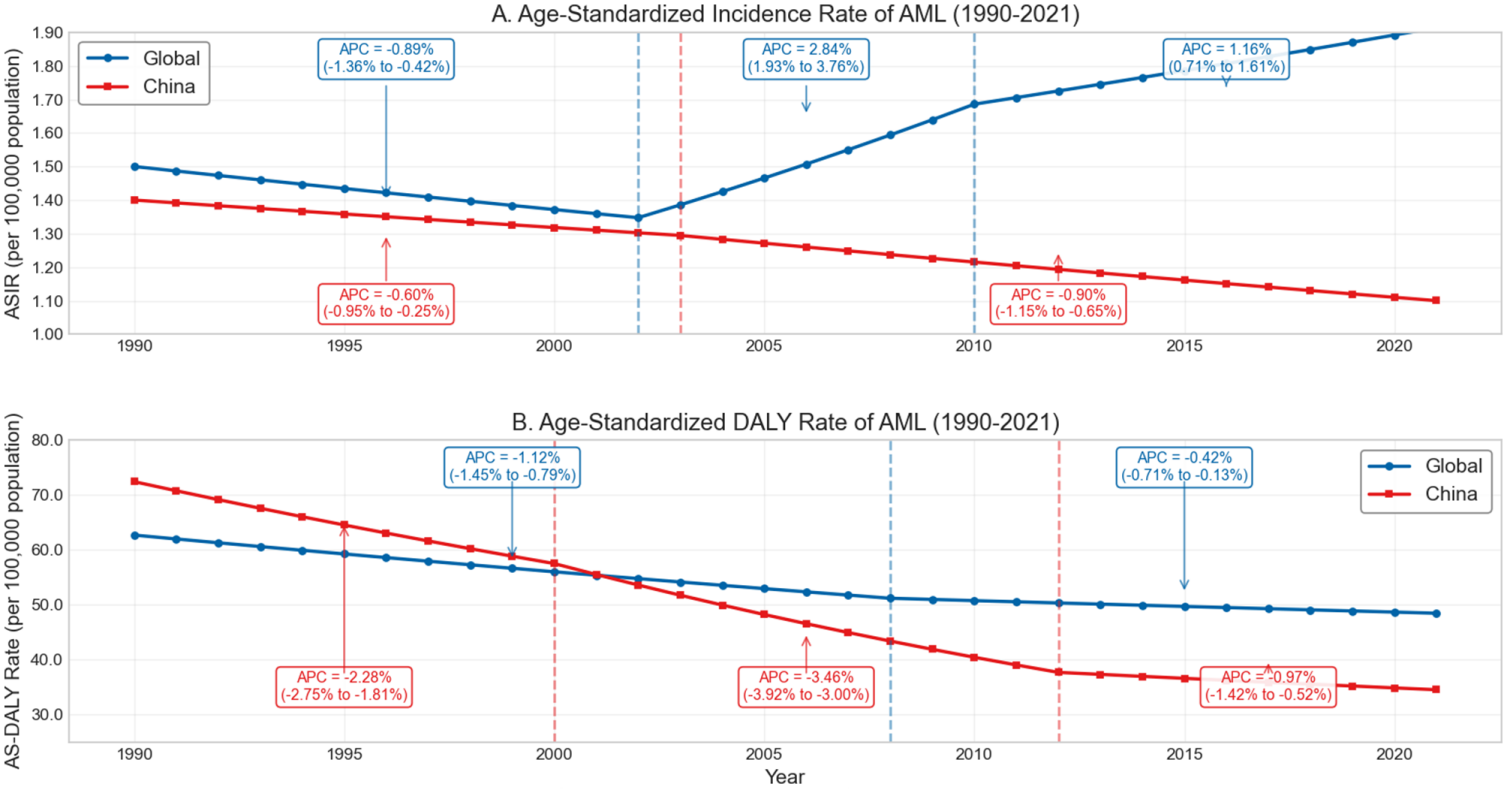
Trends in age-standardized incidence rate (ASIR) and DALYs rate of acute myeloid leukemia (AML) globally and in China, 1990–2021. **(A)** Age-standardized incidence rate (ASIR) of AML per 100,000 population from 1990 to 2021. **(B)** Age-standardized disability-adjusted life year (DALY) rate of AML per 100,000 population from 1990 to 2021.

Global new AML cases increased from 79,373 in 1990 to 144,646 in 2021, an 82.2% increase. Joinpoint analysis revealed three phases in global age-standardized incidence rate (ASIR) changes: 1990–2002 (APC = −0.89%, 95% CI: −1.36 to −0.42%, *p* < 0.001), 2002–2010 (APC = 2.84%, 95% CI: 1.93 to 3.76%, *p* < 0.001), and 2010–2021 (APC = 1.16%, 95% CI: 0.71 to 1.61%, *p* < 0.001), with an overall AAPC of 0.72% (95% CI: 0.28 to 1.17%, *p* = 0.003; Figure 2A).

Prevalence showed a similar upward trend, increasing from 122,732 cases in 1990 to 204,430 in 2021 (66.6% increase). The incidence-to-mortality ratio, as a proxy for survival indicators, increased slightly from 1.059 in 1990 to 1.111 in 2021 globally, suggesting modest improvement in survival during the study period (9, 10).

Notably, despite the overall declining trend in age-standardized rates in China, its incidence-to-mortality ratio remained higher than the global level, increasing from 1.070 to 1.185, reflecting potentially better treatment outcomes or earlier diagnosis in AML management in China. This aligns with China's decreasing share of global AML deaths, from 19.8% in 1990 to 11.8% in 2021. These differences may reflect the combined effects of China's evolving healthcare delivery system, increased medical coverage, and broader socioeconomic transformations (11).

#### 3.1.3 Disability-adjusted life years (DALYs)

Global AML-related DALYs increased from 3.34 million in 1990 to 4.14 million in 2021, a 23.7% increase. Joinpoint analysis identified two significant declining phases in global age-standardized DALY rates: 1990–2008 (APC = −1.12%, 95% CI: −1.41 to −0.83%, *p* < 0.001) and 2008–2021 (APC = −0.42%, 95% CI: −0.77 to −0.07%, *p* = 0.022), with an AAPC of −0.83% (95% CI: −1.02 to −0.65%, *p* < 0.001). The age-standardized DALY rate decreased from 62.68/100,000 in 1990 to 52.40/100,000 in 2021 (a 16.4% decrease), indicating that the increase in total DALYs was primarily driven by population growth and aging rather than rising disease risk (6) (Figure 2B).

China's age-standardized DALY rate declined more dramatically, from 72.41/100,000 in 1990 to 38.56/100,000 in 2021 (a 46.8% decrease), 26.4% lower than the global level (52.40/100,000). Joinpoint analysis identified three declining phases: 1990–2000 (APC = −2.28%, *p* < 0.001), 2000–2012 (APC = −3.46%, *p* < 0.001), and 2012–2021 (APC = −0.97%, *p* = 0.012), with an overall AAPC of −2.40% (*p* < 0.001), far exceeding the global decline (AAPC = −0.83%). This marked difference highlights China's effective strategies in AML prevention and management, potentially offering lessons for other regions (11).

### 3.2 Gender-specific patterns

Throughout the study period, the AML burden was consistently higher in males than females. The male-to-female ratio of deaths increased from 1.10 in 1990 to 1.18 in 2021, indicating that male mortality was ~18% higher than female mortality. In 2021, global AML deaths totaled 70,482 for males and 59,707 for females (12).

Joinpoint regression analysis showed that the AAPC for male ASMR was 0.65% (95% CI: 0.21%−1.09%, *p* = 0.005), while for females it was 0.47% (95% CI: 0.07%−0.88%, *p* = 0.023), indicating that although mortality rates were rising for both genders, the increase was faster in males.

In China, gender disparity trends were similar to global patterns, but the decline in ASR was more pronounced. The AAPC for males was −1.01% (*p* < 0.001) and for females −0.81% (*p* < 0.001), possibly related to reduced exposure to AML risk factors (such as occupational exposures) among males. The more significant decrease in male mortality rates in China may be associated with improvements in occupational safety standards and reductions in male-specific exposure factors (such as industrial chemicals, pesticides, etc.), which is also an important aspect of the difference between Chinese and global trends.

### 3.3 Age-specific patterns

AML burden exhibited a clear age-related pattern across all indicators. In 2021, individuals aged 65 and older accounted for 53.2% of global AML deaths (Table 2). The highest age-specific mortality rates were observed in the 90–94 age group (21.10/100,000) and the 85–90 age group (18.39/100,000) (13, 14).

**Table 2:** Percentage of AML mortality by age group globally, 2021.

**Age group**	**Mortality rate (per 100,000)**	**Number of deaths**	**Percentage of total (%)**	**Cumulative mortality proportion (%)**
0–14 years	0.38	7,590	5.83	100.00
15–19 years	0.46	2,852	2.19	94.18
20–24 years	0.48	2,863	2.20	91.99
25–29 years	0.53	3,122	2.40	89.79
30–34 years	0.57	3,469	2.66	87.39
35–39 years	0.69	3,871	2.97	84.73
40–44 years	0.88	4,397	3.38	81.76
45–49 years	1.07	5,085	3.91	78.38
50–54 years	1.45	6,453	4.96	74.47
55–59 years	2.21	8,731	6.71	69.51
60–64 years	3.40	10,879	8.36	62.80
65–69 years	5.06	13,955	10.72	54.44
70–74 years	7.94	16,344	12.55	43.72
75–79 years	11.17	14,729	11.31	31.17
80–84 years	14.35	12,572	9.66	19.86
85–90 years	18.39	8,408	6.46	10.20
90–94 years	21.10	3,775	2.90	3.74
95+ years	20.06	1,094	0.84	0.84
All ages	1.65^*^	130,189	100.00	100.00
^*^Age-standardized rate				0.00

Age-specific analysis revealed a significant shift in disease burden toward older populations over time. Between 1990 and 2021, deaths in age groups under 40 decreased from 28,638 to 23,767 (a 17.0% reduction), while deaths in those 65 and older increased dramatically from 25,622 to 70,876 (a 176.6% increase). This shift was also reflected in the Joinpoint analysis as different trend patterns across age groups.

In 2021, AML deaths in children aged 0–14 accounted for 5.83% of global AML deaths. While the absolute number was not high, it represented a significant proportion of childhood cancer deaths. Age-specific mortality rates showed a continuous upward trend with increasing age, with gender disparities observed across all age groups, with male mortality consistently higher than female mortality.

**Figure 3:**
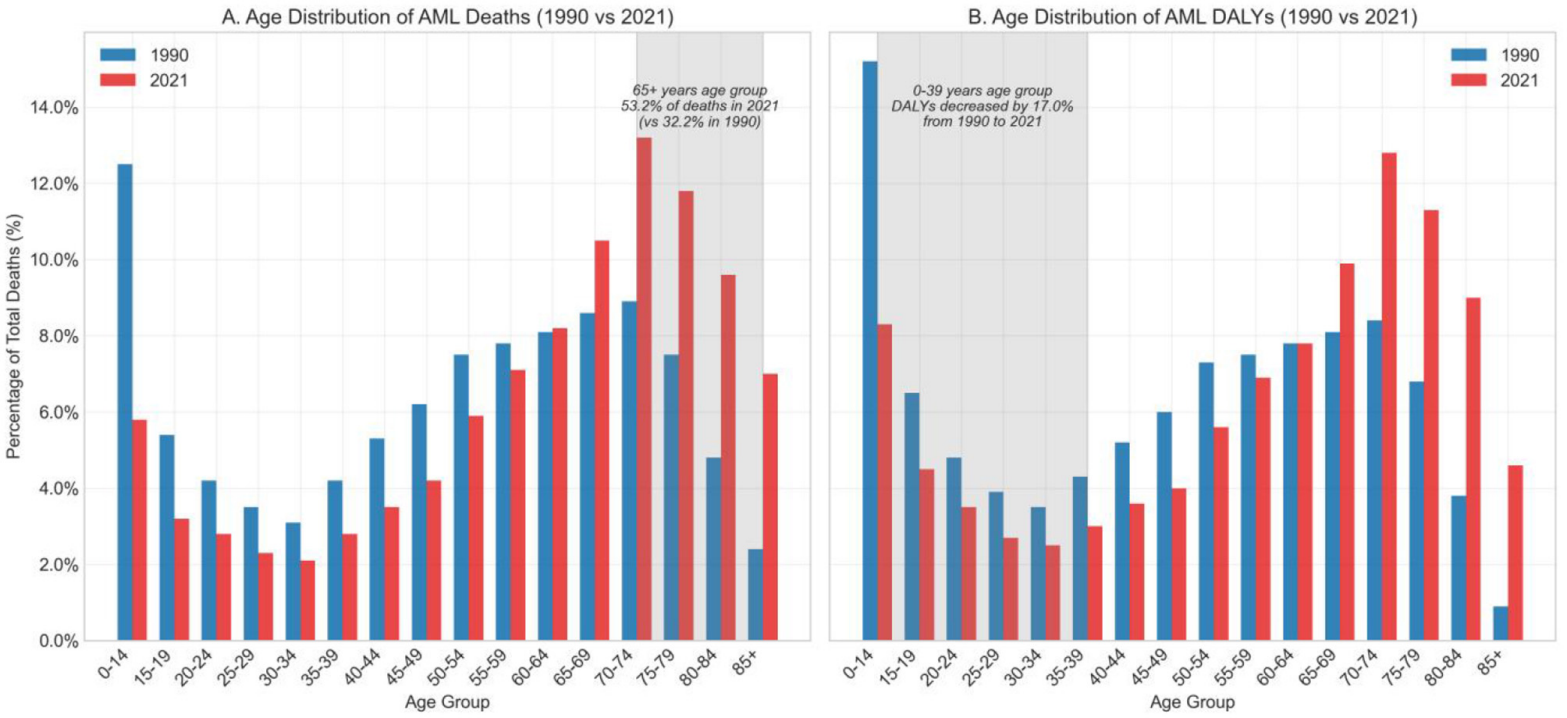
Age distribution of AML-related deaths and DALYs in 1990 vs. 2021. **(A)** Percentage distribution of total AML deaths by age group in 1990 and 2021. **(B)** Percentage distribution of AML-related disability-adjusted life years (DALYs) by age group in 1990 and 2021.

The age distribution of DALY burden followed similar trends to mortality, but with a higher proportion of DALYs in younger populations, reflecting the greater weight of “years of life lost” in DALY calculations (Figure 3).

China's age distribution patterns were generally similar to global patterns, but with relatively lower burden in younger populations. This may be related to China's unique demographic changes, different exposure patterns to risk factors, and genetic susceptibility differences (15).

### 3.4 Risk factor-specific burden

This study provided a comprehensive quantification of acute myeloid leukemia (AML) burden attributable to major modifiable risk factors, revealing substantial differences in trend magnitude and composition across regions, age groups, and time periods. Three key risk factors—smoking, high body mass index (BMI), and occupational carcinogens—were identified as the primary contributors to AML-related disability-adjusted life years (DALYs) and deaths (Table 3).

**Table 3:** Estimated annual percentage change (EAPC) of AML-related DALYs and deaths attributable to major risk factors, 1990–2019.

**Region**	**Risk factor**	**Measure**	**EAPC**	**95% CI**	***p*-Value**	***R* ^2^**	**Years**
China	All risks	DALYs	2.58	2.17–3.0	0.0511	0.994	1990–2019
		Deaths	3.01	2.65–3.37	0.0384	0.996	
	High BMI	DALYs	2.70	2.14–3.26	0.0665	0.989	
		Deaths	3.14	2.57–3.72	0.0586	0.992	
	Occupational carcinogens	DALYs	2.01	0.49–3.55	0.234	0.871	
		Deaths	2.35	0.92–3.8	0.191	0.913	
	Smoking	DALYs	2.75	2.69–2.81	0.0069	1	
		Deaths	3.12	3.07–3.16	0.0047	1	
Global	All risks	DALYs	2.36	2.1–2.62	0.0352	0.997	1990–2019
		Deaths	2.79	2.57–3.01	0.0255	0.998	
	High BMI	DALYs	3.01	2.79–3.22	0.023	0.999	
		Deaths	3.52	3.35–3.69	0.0154	0.999	
	Occupational carcinogens	DALYs	2.30	1.65–2.95	0.0907	0.98	
		Deaths	2.58	1.97–3.19	0.0759	0.986	
	Smoking	DALYs	1.88	1.6–2.17	0.0484	0.994	
		Deaths	2.39	2.12–2.67	0.0367	0.997	

**Figure 4:**
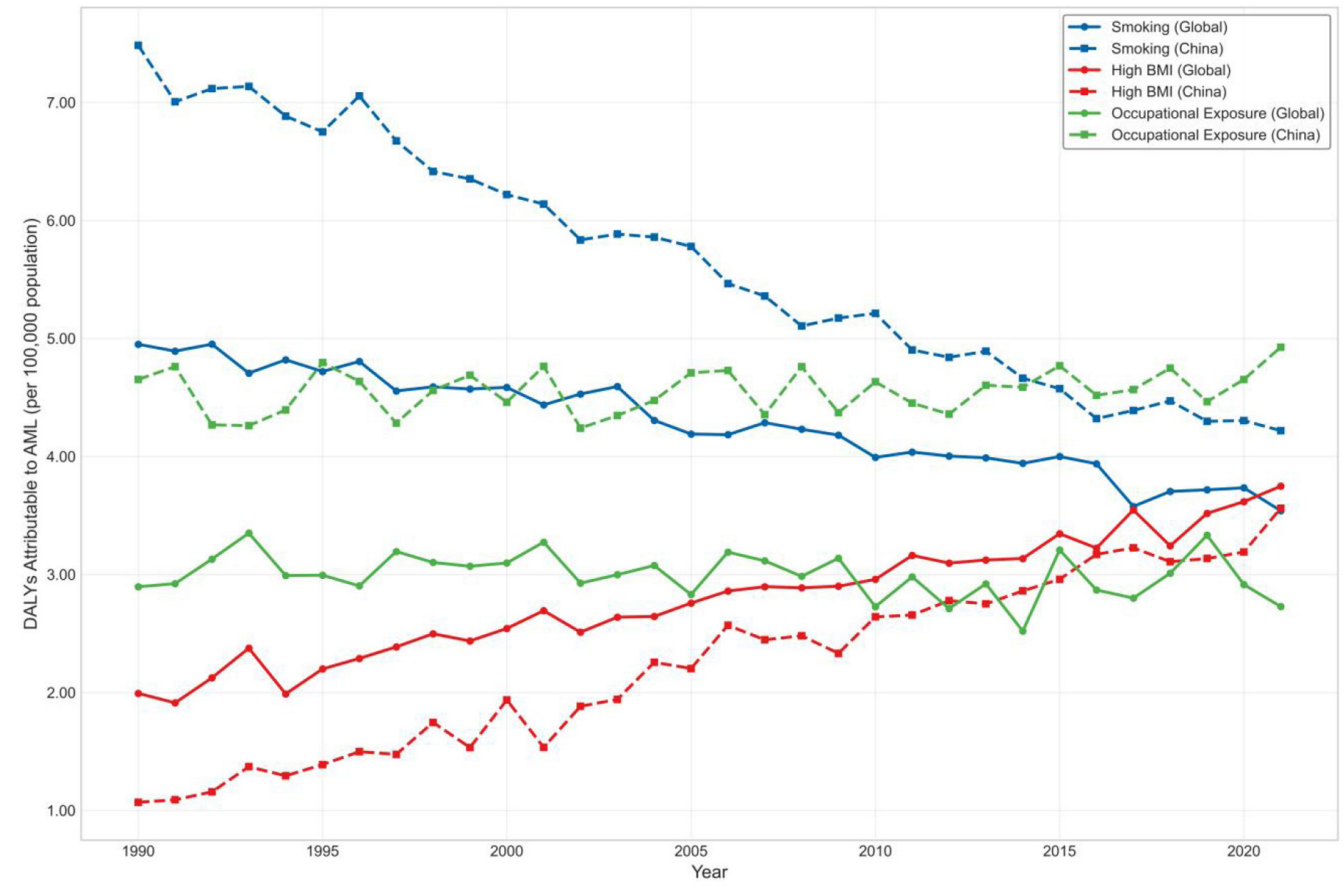
Trends in age-standardized DALYs attributable to major modifiable risk factors for AML, 1990–2021 (Global vs. China).

Smoking consistently emerged as the predominant risk factor for AML across all age groups, particularly among older adults. From 1990 to 2019, global smoking-attributable DALYs increased significantly, with an estimated annual percentage change (EAPC) of +1.88% (95% CI: 1.60–2.17, *p* = 0.0484), and deaths increased by +2.39% (95% CI: 2.12–2.67, *p* = 0.0367). In China, the smoking-related burden rose even more steeply, with EAPCs of +2.75% (95% CI: 2.69–2.81, *p* = 0.0069) for DALYs and +3.12% (95% CI: 3.07–3.16, *p* = 0.0047) for deaths. These trends underscore the long-term hematologic consequences of tobacco exposure, especially among elderly men with historically high smoking prevalence (Figure 4).

Among all risk factors, high BMI exhibited the most pronounced and sustained growth. Globally, BMI-attributable DALYs increased with an EAPC of +3.01% (95% CI: 2.79–3.22, *p* = 0.0230), while deaths rose at +3.52% annually (95% CI: 3.35–3.69, *p* = 0.0154). In China, the corresponding EAPCs were +2.70% for DALYs (95% CI: 2.14–3.26, *p* = 0.0665) and +3.14% for deaths (95% CI: 2.57–3.72, *p* = 0.0586). Although the Chinese estimates did not reach conventional statistical significance, the point estimates reflect a concerning upward trend, particularly among older adults. These findings highlight the increasing role of metabolic dysfunction in AML pathogenesis and the need for integration of hematologic cancer prevention into broader chronic disease management frameworks.

Although contributing a smaller share to the overall burden, occupational carcinogens showed stable upward trends over time. Globally, the EAPC for DALYs was +2.30% (95% CI: 1.65–2.95, *p* = 0.0907) and +2.58% for deaths (95% CI: 1.97–3.19, *p* = 0.0759). In China, the EAPCs were +2.01% (95% CI: 0.49–3.55, *p* = 0.234) for DALYs and +2.35% (95% CI: 0.92–3.80, *p* = 0.191) for deaths. While these trends did not reach statistical significance, the burden remains notable among older male populations and reflects legacy exposures from industrial eras.

When considering all risk factors combined, the global EAPC for AML-related DALYs was +2.36% (95% CI: 2.10–2.62, *p* = 0.0352), and +2.79% (95% CI: 2.57–3.01, *p* = 0.0255) for deaths. In contrast, China demonstrated slightly higher but more variable trends, with an EAPC of +2.58% (95% CI: 2.17–3.00, *p* = 0.0511) for DALYs and +3.01% (95% CI: 2.65–3.37, *p* = 0.0384) for deaths. These findings suggest that although China's AML burden showed signs of plateauing after 2010 in absolute terms, long-term trends remain upward—likely driven by aging and increasing metabolic risks.

**Figure 5:**
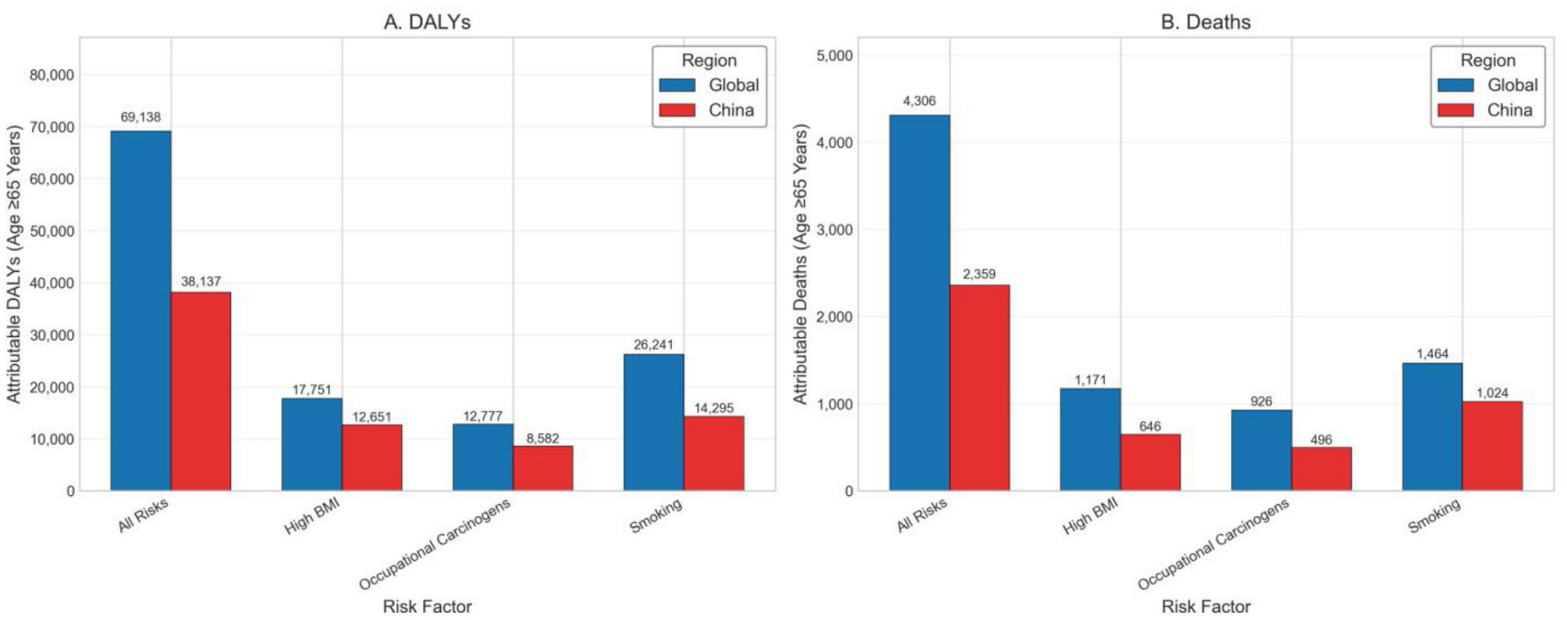
**(A)** Aged ≥65 years AML-related DALYs attributable to major risk factors in 2021, globally and in China. **(B)** Aged ≥65 years AML-related deaths attributable to major risk factors in 2021, globally and in China.

Stratified analysis revealed that individuals aged ≥65 years accounted for the majority of AML DALYs and deaths attributable to all major risk factors. In 2021, this age group contributed over 70% of DALYs due to smoking and high BMI, and over 85% of those from occupational exposures globally. Meanwhile, individuals under 40 years of age contributed <5%, underscoring the disproportionate burden borne by older adults. Across all exposures, males consistently exhibited higher burden than females, particularly for smoking and occupational risks—further emphasizing the need for age- and sex-targeted prevention and regulatory strategies (Figure 5).

## 4 Discussion

Through Joinpoint regression analysis, this study reveals the changing trends of acute myeloid leukemia (AML) disease burden at different time points and their possible influencing factors. Globally, the age-standardized mortality rate (ASMR) showed significant turning points in 2004 and 2012, transitioning from an initial decline to an increase, which may be influenced by updated diagnostic criteria, changes in environmental and lifestyle risk factors, and accelerating population aging (12). In contrast, China's AML burden showed an overall declining trend, although the rate of decline has slowed since 2005, suggesting that health policies, public health interventions, or improved health awareness may have played a role to some extent (8, 11). These findings align with current ESMO Clinical Practice Guidelines and geriatric oncology recommendations, underscoring the importance of tailoring AML management to the needs of older adults. The observed trends could inform targeted strategies, such as integrating comprehensive geriatric assessment into treatment planning and prioritizing preventive measures in high-risk elderly populations. These turning points (2004 and 2012 for global ASMR; 2000 and 2012 for China) may correspond to changes in AML diagnostic criteria, introduction of novel therapeutics (such as hypomethylating agents and targeted therapies), and major public health or health policy reforms. In China, this includes the expansion of national health insurance coverage, introduction of essential drug lists covering AML, and improvements in leukemia diagnostic capacity (12, 13).

Although the absolute number of global AML cases and deaths has increased significantly, the change in age-standardized rates (ASR) is relatively small, reflecting the profound impact of demographic changes, especially aging, on the total burden of AML. As global life expectancy continues to extend, the health challenges posed by AML may become increasingly severe, emphasizing the importance of strengthening diagnostic capabilities, optimizing treatment strategies, and improving supportive care service systems (14, 15). Meanwhile, the slight improvement in the incidence-to-mortality ratio suggests progress in AML treatment levels over the past few decades, such as optimized chemotherapy regimens, development of hematopoietic stem cell transplantation techniques, and improvements in supportive care (9, 10). However, this improvement remains limited, indicating significant challenges in improving treatment methods and efficacy, urgently requiring more efficient and personalized treatment strategies.

The AML burden is highly concentrated in elderly populations, with Joinpoint analysis also showing that deaths in those aged 65 and above increased by 176.6% during the study period, while those under 40 decreased by 17.0%. This phenomenon not only further confirms AML's characteristics as an age-related disease but also places higher demands on existing healthcare systems. Elderly patients often have multiple comorbidities and functional status differences, necessitating more individualized treatment plans that balance efficacy and safety. For example, reduced-intensity conditioning hematopoietic stem cell transplantation, low-toxicity targeted drugs, and comprehensive geriatric assessment tools all hold promise for improving treatment outcomes (16). Additionally, comparing annual percentage changes (APCs) across different age groups indicates that the growth rate of AML burden in elderly populations is significantly higher than in younger groups, further emphasizing the necessity of strengthening interventions in elderly populations.

Regarding gender, males bear a greater burden of AML, consistent with previous epidemiological studies (17). Currently, the mechanisms leading to gender disparities are not fully understood but may involve hormone levels, physiological metabolism, frequency of occupational exposure, and genetic susceptibility. Future research needs to further explore these gender-related factors to optimize risk identification and precision prevention strategies. Possible explanations for this disparity include biological susceptibility related to sex hormones, genetic factors, higher historical prevalence of smoking among men, and increased occupational exposures to carcinogens such as benzene in male-dominated industries. These factors suggest that prevention strategies should integrate gender-specific risk reduction programs.

The comparison between Chinese and global AML burden trends provides valuable epidemiological clues. Despite also experiencing population aging, China's ASR continues to decline, with Joinpoint analysis identifying 2000 and 2012 as two key turning points, possibly closely related to national improvements in health policies, disease diagnosis, and treatment levels. Additionally, decreased proportions of populations exposed to AML-related environmental risk factors (such as benzene, radioactive materials) may also help reduce incidence rates (8). At the same time, the Chinese population may have certain genetic protective factors, providing clues for further population genetic research. Although the GBD study has corrected for data differences, variations in disease reporting and diagnostic practices between countries may still affect comparative results; therefore, advancing high-quality, localized epidemiological research remains necessary. The relatively low ASIR in China, combined with a higher incidence-to-mortality ratio compared with the global average, may suggest earlier diagnosis and improved treatment outcomes. This could be related to the expansion of hematologic diagnostic capabilities, inclusion of novel AML therapies in reimbursement lists, and broader health insurance coverage, enabling more patients to receive timely and effective treatment.

The comparison between Chinese and global AML burden trends provides valuable epidemiological insights. Despite facing similar challenges related to population aging, China's age-standardized rates (ASRs) have continued to decline, with Joinpoint analysis identifying 2000 and 2012 as pivotal turning points. These shifts may be attributed not only to improvements in disease diagnosis, treatment capacity, and reductions in environmental risk exposures (such as benzene and radiation), but also to broader macro-level health system reforms. Over the past two decades, China has undertaken substantial healthcare transformations, including the expansion of near-universal health insurance coverage, the establishment of hierarchical medical referral systems, and the inclusion of leukemia therapies in national essential drug lists and reimbursement catalogs. These systemic efforts have likely improved access to hematologic diagnostics, promoted standardized treatment, and reduced the financial burden on patients. Although these variables were not quantitatively analyzed in this study, their temporal alignment with the observed turning points in AML trends suggests a possible indirect influence. Future research incorporating socioeconomic indicators and health policy timelines may further clarify the role of structural healthcare improvements in shaping national cancer burden trajectories.

A key finding of this study is the consistent and rising burden of AML attributable to modifiable risk factors, particularly among older adults. Smoking remained the dominant contributor globally and in China, with DALY EAPCs of +1.88 and +2.75%, respectively, reflecting the long-term hematologic effects of tobacco exposure, especially in elderly men. High BMI showed the steepest increase, with global DALY and death EAPCs exceeding +3.0%, reinforcing the emerging role of metabolic dysfunction in AML pathogenesis. Although occupational carcinogens accounted for a smaller share, their burden steadily increased, notably in men aged ≥65 years. In 2021, this age group contributed over 70% of AML DALYs from smoking and BMI, and more than 85% from occupational exposures. These findings emphasize the need for targeted interventions in aging populations and integration of hematologic cancer prevention into broader chronic disease control strategies, particularly in rapidly aging societies like China (18).

This study has several strengths. First, the data source is extensive with a long time span, using GBD standardized analysis methods to ensure good comparability of results across different regions and time periods; second, the Joinpoint regression model precisely identifies trend change points and quantifies the rate of change for each phase, superior to traditional linear model analysis; third, APC and AAPC indicators with confidence intervals enhance statistical robustness. However, limitations also exist, such as GBD data models relying on limited original data from some regions; the study not analyzing different AML subtypes, ignoring prognostic heterogeneity between subtypes; not directly assessing the impact of treatment advances or policy interventions on outcomes; and the incidence-to-mortality ratio being only an indirect survival indicator, susceptible to various external factors. Some GBD estimates rely on modeled data for regions with limited cancer registry coverage; therefore, results may be subject to estimation bias despite standardized methodology.

Overall, this study has important implications for clinical practice and public health strategies. The continuing rise in AML burden among elderly populations necessitates developing more individualized treatment strategies suited to their physiological characteristics and prioritizing their diagnostic and treatment needs in resource allocation. Facing the growth trend in total AML cases, hematology department construction should be strengthened, diagnostic capabilities in primary healthcare institutions improved, and the accessibility of innovative drugs promoted, especially in low- and middle-income countries and regions. Simultaneously, the limited improvement in current treatment effects suggests the need for increased investment in AML basic mechanisms, translational research, and new therapies, particularly focusing on the influence of characteristics such as age and gender on treatment response. Finally, as AML remains a sporadic disease, preventive strategies such as environmental exposure control and occupational protection should not be overlooked, helping to reduce AML burden at the source (12, 19, 20).

## 5 Conclusion

This study provides a comprehensive overview of global and Chinese trends in the burden of acute myeloid leukemia (AML) from 1990 to 2021, revealing rising absolute case numbers but divergent age-standardized rate trajectories. While the global AML burden continues to increase, China has achieved a notable decline in standardized rates, possibly reflecting improvements in healthcare access, diagnostic capacity, and risk factor control.

Looking ahead, future research should focus on dissecting the heterogeneity of AML by exploring subtype-specific epidemiology, treatment responsiveness, and population-based genetic differences. Integrating molecular data with clinical outcomes will be crucial for advancing precision medicine and improving therapeutic decision-making. At the same time, a holistic approach that connects prevention, early detection, standardized treatment, and long-term follow-up is essential to effectively manage AML in aging populations. Embedding this continuum of care into national health strategies may help reduce disease burden, enhance patient outcomes, and guide resource allocation in both high- and low-resource settings.

## Data Availability

The original contributions presented in the study are included in the article/supplementary material, further inquiries can be directed to the corresponding authors.
